# CXCR7 promotes migration and invasion in head and neck squamous cell carcinoma by upregulating TGF-β1/Smad2/3 signaling

**DOI:** 10.1038/s41598-019-54705-x

**Published:** 2019-12-02

**Authors:** Nayoung Kim, Hyewon Ryu, Solbi Kim, Mina Joo, Heung Jin Jeon, Myung-Won Lee, Ik-Chan Song, Mi-Na Kim, Jin-Man Kim, Hyo Jin Lee

**Affiliations:** 1 Department of Medical Science, Chungnam National University College of Medicine, Daejeon, 35015 Korea; 2 Infection Control Convergence Research Center, Chungnam National University College of Medicine, Daejeon, 35015 Korea; 3 Department of Internal Medicine, Chungnam National University College of Medicine, Daejeon, 35015 Korea; 4 Cancer Research Institute, Chungnam National University, Daejeon, 35015 Korea; 5 Department of Pathology, Chungnam National University College of Medicine, Daejeon, 35015 Korea

**Keywords:** Head and neck cancer, Oncogenes

## Abstract

The chemokine receptor CXCR7 has been suggested to play important roles in the progression of several types of cancers. However, few studies have investigated the biological roles of CXCR7 in head and neck squamous cell carcinoma (HNSCC). CXCR7 expression and its clinical implications were examined in 103 HNSCC tissues using immunohistochemistry (IHC). The biological roles and mechanisms of CXCR7-mediated signaling pathways were investigated in HNSCC cells through CXCR7 overexpression *in vitro* and *in vivo*. High expression of CXCR7 was significantly associated with tumor size (*P* = 0.007), lymph node metastasis (*P* = 0.004), and stage (*P* = 0.020) in HNSCC. Overexpression of CXCR7 in HNSCC cells enhanced cell migration and invasion *in vitro* and promoted lymph node metastasis *in vivo*. CXCR7 also induced epithelial–mesenchymal transition through PI3K/AKT. CXCR7 increased secretion of transforming growth factor-β1 (TGF-β1) and promoted EMT through phosphorylated Smad2/3. Taken together, our results provide functional and mechanistic roles of CXCR7 as a master regulator of oncogenic TGF-β1/Smad2/3 signaling in HNSCC, suggesting that CXCR7 might be a therapeutic target for the treatment of HNSCC.

## Introduction

Head and neck squamous cell cancer (HNSCC) constitutes a heterogeneous group of cancers. HNSCC is an epithelial malignancy with primary sites in the lip, oral cavity, pharynx, larynx, and paranasal sinuses^1,2^. High cure rates are achieved for localized HNSCC using surgery, radiation, and chemoradiation. However, recurrence after curative resection is common, and survival rates for recurrent/metastatic disease remain poor, with a 10% 5-year overall survival rate^3^. Therefore, an understanding of the molecular mechanisms of cancer progression is necessary to advance the treatment of HNSCC.

In the tumor microenvironment, chemokine signaling systems play critical roles in tumor progression, invasion, migration, and metastasis^4^. Chemokines and chemokine receptors are differentially expressed in various malignant tumors^5,6^. Growing evidence shows that CXCR7 plays a crucial role in the development of tumors^7^. Furthermore, upregulation of CXCR7 serves as an oncogene in various cancers, such as breast and lung cancer^5,8^.

Tumors metastasize through decreased cell adhesion, basement membrane perforation, migration by circulation, immune escape, and formation of colonies at distant sites^9^. Epithelial–mesenchymal transition (EMT) is essential for initiation and progression of metastasis^10,11^. Transforming growth factor (TGF)-β signaling is known to induce EMT through various intracellular messengers. Recent studies have shown that TGF-β promotes tumor progression and metastasis by regulating chemokines or chemokine receptors in the tumor microenvironment^12–14^. However, little is known concerning the role of CXCR7 and TGF-β in HNSCC. Thus, we performed this study to investigate the biologic functions of CXCR7 and its effects on tumor growth and progression in HNSCC.

## Results

### Expression of CXCR7 and its association with clinicopathological features

**Figure 1:**
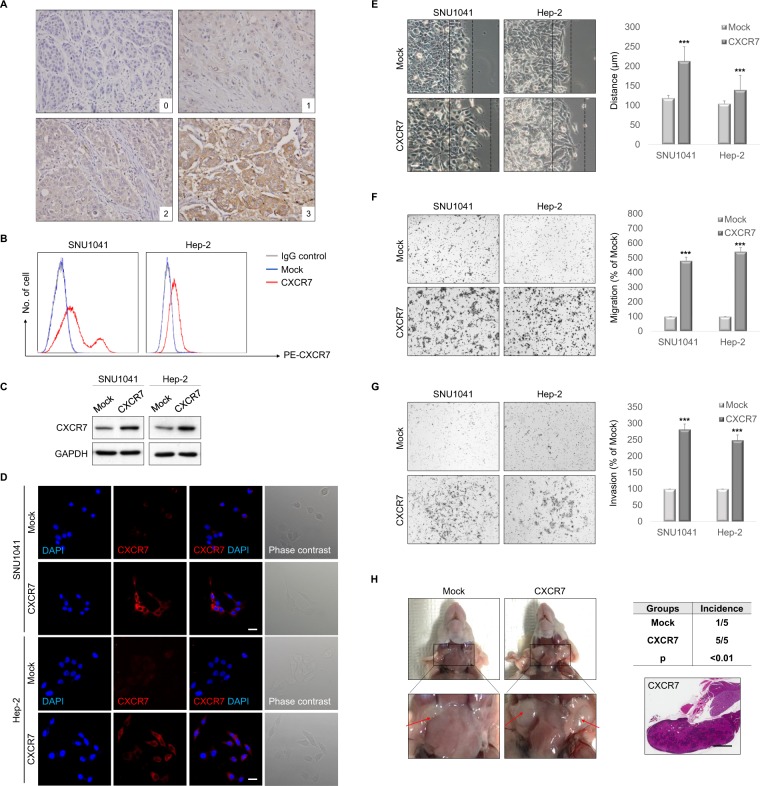
CXCR7 enhanced cell migration and invasion *in vitro* and *in vivo*. (**A**) Expression of CXCR7 protein in HNSCC tissues. HNSCC tissues were immunohistochemically stained with an anti-CXCR7 antibody (x400). 0, no staining; 1, weak staining; 2, intermediate staining; 3, strong staining. (**B**) Exogenous CXCR7 was expressed in SNU1041 and Hep-2 cells. The surface expression of CXCR7 was evaluated by FACS analysis using a phycoerythrin (PE)-anti-CXCR7 monoclonal antibody to detect CXCR7 expression; a matched PE mouse IgG served as the isotype control. (**C**) Western blot analysis showing increased expression of CXCR7 in CXCR7-overexpressed cells compared with mock cells. Full-length blots are presented in Supplementary Fig. S3. (**D**) Immunofluorescence visualization of CXCR7 and phase-contrast microscopic images. Immunofluorescence staining showed an upregulated expression of CXCR7. Scale bars, 20 μm. **(E)** Wound-healing assay indicating CXCR7 overexpression enhanced cell motility (x100). Scale bars, 100 μm. (**F**,**G**) Transwell migration and invasion assay showing that CXCR7 overexpression promoted cell migration and invasion. (**H**) SNU1041-mock- or SNU1041-CXCR7-overexpressed cells were injected into the tongue of mice to test lymph node metastasis capacity (left). H&E staining of lymph node sections from the CXCR7 overexpression implanted group (right). Scale bars, 1 mm. ***P < 0.001.

To analyze the function of CXCR7 in HNSCC progression, we first evaluated CXCR7 expression by immunohistochemical analysis of tumor specimens from 103 patients with HNSCC. CXCR7 was located in the membrane and/or cytoplasm, and the intensity of the immunohistochemical staining varied as follows: negative staining (score 0), 17 cases; weak staining (score 1), 34 cases; moderate staining (score 2), 38 cases; strong staining (score 3), 14 cases (Fig. 1A). Of the 103 tumors examined, 51 (49.5%) were classified as CXCR7-low and 52 (50.5%) were classified as CXCR7-high tumors. We next analyzed the correlation between CXCR7 expression and various clinicopathological factors that can affect the prognosis of patients with HNSCC. The results are summarized in Table 1. The CXCR7-high group had increased tumor size (*P* = 0.007), lymph node metastasis (*P* = 0.004), and advanced tumor stage (*P* = 0.020). These findings indicate that high CXCR7 expression is strongly associated with advanced disease in HNSCC.

**Table 1:** Relationship of CXCR7 expression and clinicopathological characteristics in patients with head and neck squamous cell carcinoma.

Variable	Total n = 103	CXCR7		
		Low (n = 51)	High (n = 52)	*P*-value
**Age**				
<65	59	27 (52.9%)	24 (46.2%)	0.491*
≥65	44	24 (47.1%)	28(53.8%)	
**Gender**				
Male	93	43 (84.3%)	50 (96.2%)	0.052*
Female	10	8 (15.7%)	2 (3.8%)	
**ECOG PS**				
0/1	98	49 (96.1%)	49 (94.2%)	1.000*
2	5	2 (3.9%)	3 (5.8%)	
**Smoking**				
Never	13	8 (15.7%)	5 (9.6%)	0.497^†^
Current	20	11 (21.6%)	9 (17.3%)	
Former	70	32 (62.7%)	38 (73.1%)	
**Tumor size**				
T1/2	59	36 (70.6%)	23 (44.2%)	0.007*
T3/4	44	15 (29.4%)	29 (55.8%)	
**Lymph node metastasis**				
Negative	46	30 (58.8%)	16 (30.8%)	0.004*
Positive	57	21 (41.2%)	36 (69.2%)	
**Stage**				
I	19	15 (29.4%)	4 (7.7%)	0.020^†^
II	16	9 (17.6%)	7 (13.5%)	
III	15	5 (9.8%)	10 (19.2%)	
IV	53	22 (43.1%)	31 (59.6%)	

ECOG PS, Eastern Cooperative Oncology Group performance status

^*^P values were calculated by pairwise comparisons from χ 2 test.

^†^*P* values were calculated by comparisons of groups from linear-by-linear associations.

### CXCR7 promotes cell migration and invasion *in vitro* and *in vivo*

To investigate whether CXCR7 promotes tumor progression of HNSCC, we created stable HNSCC cells with overexpression of CXCR7. The relative expression of CXCR7 in two different HNSCC cell lines, SNU1041 and Hep-2, was confirmed by flow cytometry, Western blotting, and immunofluorescence staining (Fig. 1B–D). Overexpression of CXCR7 significantly increased motility, migration, and invasion of HNSCC cells (Fig. 1E–G). To explore further the effect of CXCR7 overexpression on tumor metastasis in HNSCC *in vivo*, HNSCC cells overexpressing CXCR7 were implanted into the tongue of BALB/c nude mice. Eight weeks after cell inoculation, cervical lymph node metastasis was significantly increased in mouse xenografts with CXCR7 overexpression compared with controls (Fig. 1H). These results suggest that CXCR7 promotes LN metastasis of HNSCC cells through increased cell motility and invasion.

### CXCR7 induces EMT through the PI3K/AKT signaling pathway

**Figure 2:**
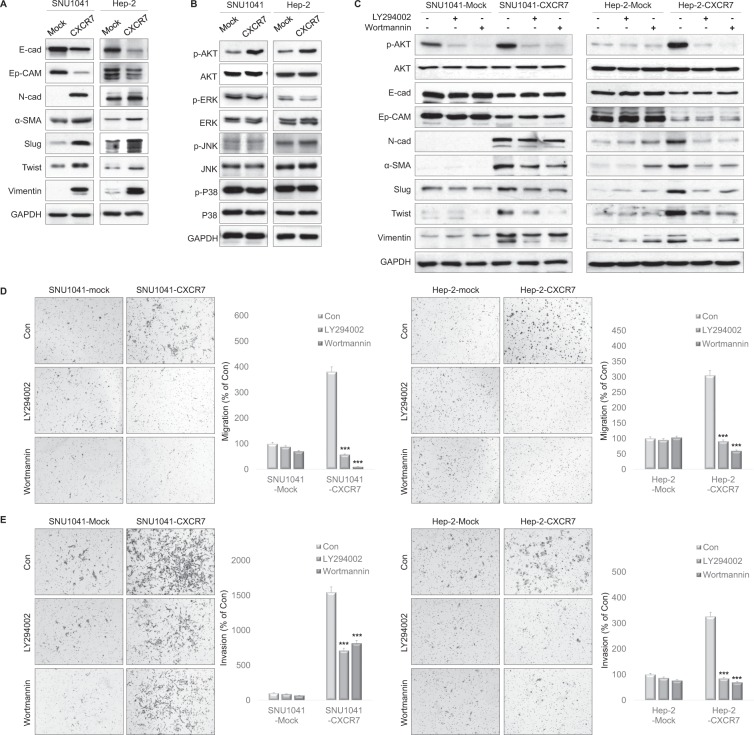
CXCR7 overexpression enhanced EMT through the AKT signaling pathway. (**A**) Western blot analysis showing decreased expression of epithelial makers, increased expression of mesenchymal markers (N-cadherin, α-SMA and Vimentin) and EMT-related transcription factors (Slug and Twist) in CXCR7-overexpressed cells compared with mock cells. Full-length blots are presented in Supplementary Fig. S3. (**B**) AKT, ERK, JNK, and P38 expression levels were determined by Western blot analysis in cells with CXCR7 overexpression. Full-length blots are presented in Supplementary Fig. S3. (**C**) Western blot analysis showed that PI3K inhibitors LY294002 and wortmannin effectively decreased the expression of p-AKT induced by CXCR7 overexpression. Inhibition of PI3K activity significantly reversed EMT markers. Mock and CXCR7-overexpressed cells were treated with 10 μM of LY294002 or 1 μM of wortmannin for 24 h. Full-length blots are presented in Supplementary Fig. S3. (**D**,**E**) Transwell migration and invasion assay showing that LY294002 and wortmannin inhibited CXCR7-induced cell migration and invasion. ***P < 0.001.

CXCR7-overexpressed cells displayed morphologic changes from their normal round-shaped, epithelial phenotype to a spindle-shaped, mesenchymal phenotype (Supplementary Fig. S1A). Moreover, the expression of epithelial markers such as E-cadherin and Ep-CAM was markedly downregulated in CXCR7-overexpressed cells, whereas the expression of mesenchymal markers including N-cadherin, α-SMA, Slug, Twist, and Vimentin was upregulated (Fig. 2A). Immunofluorescence results showed that E-cadherin was decreased and Vimentin was increased in CXCR7-overexpressed cells (Supplementary Fig. S1B)

To explore the signaling mechanisms involved in the EMT of CXCR7-overexpressed cells, we examined the AKT, ERK1/2, JNK, and p38 signaling pathways. Phosphorylation of AKT was increased in CXCR7-overexpressed cells compared to mock cells (Fig. 2B). To determine whether PI3K/AKT pathway activity was required for CXCR7-mediated EMT, mock and CXCR7-overexpressed cells were treated with the PI3K/AKT inhibitor LY294002 or wortmannin. There was no significant change in EMT markers when PI3K/AKT inhibitors were treated in mock cells (Fig. 2C). However, N-cadherin, α-SMA, Slug, Twist, and Vimentin expressions were decreased by PI3K/AKT inhibitors in CXCR7-overexpressed cells (Fig. 2C). The above results were also supported by immunofluorescence results (Supplementary Fig. S1C). Inhibition of PI3K/AKT suppressed the enhancement of cell migration and invasion, which was induced by CXCR7 overexpression (Fig. 2D,E). Taken together, these findings indicate that PI3K/AKT played a crucial role in the CXCR7-induced EMT of HNSCC cells.

### CXCR7 promotes TGF-β1 signaling and Smad2/3 phosphorylation-enhanced EMT

**Figure 3:**
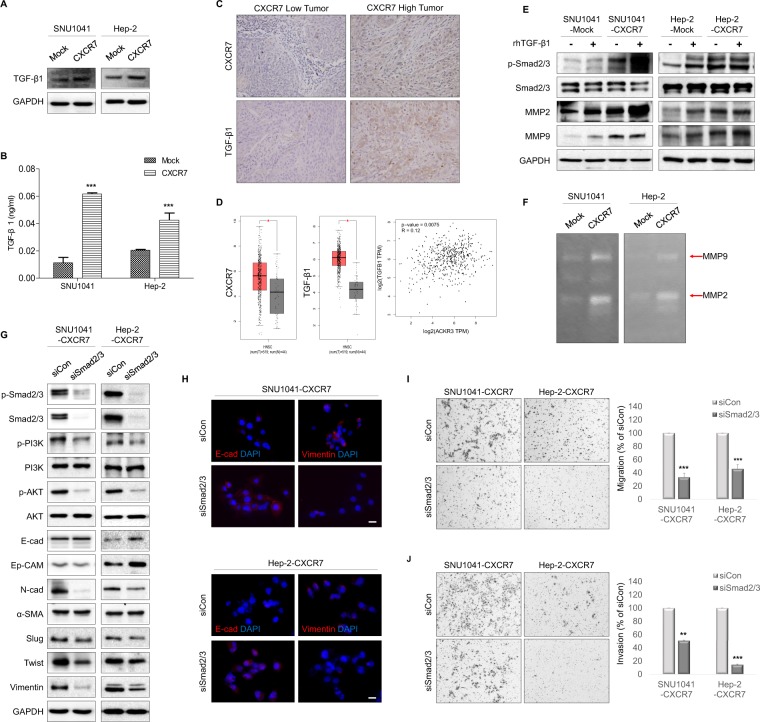
CXCR7 overexpression enhanced EMT through the TGF-β1 and Smad2/3 signaling pathways. (**A**) Western blot analysis showed increased expression of TGF-β1 in CXCR7-overexpressed cells compared with mock cells. Full-length blots are presented in Supplementary Fig. S3. (**B**) TGF-β1 levels in conditioned medium secreted by CXCR7 cells were quantified using ELISA. (**C**) TGF-β1 was highly expressed in the tissue specimen of the CXCR7-high group (x400). (**D**) Upregulation of CXCR7 and TGF- β1 in HNSCC (left and middle panels) and the correlation of CXCR7 and TGF-β1 expression (right panel) in TCGA dataset. (**E**) Western blot analysis showing increased expression of p-Smad2/3, MMP2, and MMP9 in CXCR7-overexpressed cells compared with mock cells. Mock and CXCR7-overexpressed cells were treated with 10 ng/ml of recombinant TGF-β1 for 24 h. Full-length blots are presented in Supplementary Fig. S3. (**F**) MMP2 and MMP9 activity in conditioned medium was detected by gelatin zymography assays in mock- and CXCR7-overexpressed cells. (**G**) CXCR7 cells were transfected with siRNA for Smad2/3. Knockdown of Smad2/3 significantly changed EMT markers and decreased phosphorylated AKT. Full-length blots are presented in Supplementary Fig. S3. (**H**) Immunofluorescence staining showed increased expression of E-cadherin and downregulated expression of vimentin by Smad2/3 inhibition. Scale bars, 20 μm. (**I** and **J**). Transwell migration and invasion assay showing that knockdown of Smad2/3 inhibited CXCR7-induced cell migration and invasion. **P < 0.01; ***P < 0.001.

TGF-β1, which is produced by tumor cells and immune cells, is a pleiotropic cytokine that regulates tumor progression and EMT, and is abundantly expressed in tumors^15^. In response to TGF-β, Smad2 and Smad3, which regulate target genes through interactions with other transcription factors, are activated^16^. We investigated TGF-β1-induced EMT in CXCR7-overexpressed cells. TGF-β1 is highly upregulated in CXCR7-overexpressed cells (Fig. 3A) and increased levels of TGF-β1 were detected in conditioned medium of CXCR7-overexpressed cells (Fig. 3B). In the tissue of CXCR7-high patients, there was significantly high expression of TGF-β1 (Fig. 3C). A pan-cancer analysis from The Cancer Genome Atlas (TCGA) also showed increased expression of CXCR7 and TGF-β1 in HNSCC. Moreover, there was a correlation between CXCR7 and TGF-β1 expression (Fig. 3D). We next examined whether CXCR7 overexpression affects the expression of Smad2/3, which are downstream targets of TGF-β1 signaling. The expression and phosphorylation of Smad2/3 were increased in CXCR7-overexpressed cells (Fig. 3E). In addition, treatment with recombinant TGF-β1 promoted Smad2/3 phosphorylation. Moreover, the expression of MMP2 and MMP9 was also increased in CXCR7-overexpressed cells compared to mock cells. This is known to cleave latent TGF-β1 to form active TGF-β1 (Fig. 3E,F). To confirm whether CXCR7 overexpression induces the migration and invasion of HNSCC cells in a TGF-β1 ligand dependent manner, we treated cancer cells with an anti-TGF-β1 monoclonal antibody. Neutralization of TGF-β1 suppressed the cell migration and invasion induced by CXCR7 overexpression (Supplementary Fig. S2). Therefore, the concordant expression of the CXCR7/TGF-β1 axis might highlight the importance of CXCR7 in determining the outcome of patients with cancer.

To determine whether CXCR7 overexpression induces EMT in HNSCC cells by TGF-β1/Smad2/3 signaling, we knocked down Smad2/3 expression using siRNA in CXCR7-overexpressed cells. Knockdown of Smad2/3 increased the expression of E-cadherin and Ep-CAM in Hep-2-CXCR7 cells, whereas it decreased the expression of N-cadherin, Slug, Twist, and Vimentin, as well as PI3K and AKT phosphorylation in SNU-CXCR7 and Hep-2-CXCR7 cells (Fig. 3G). These data demonstrate that TGF-β1/Smad2/3 signaling plays a crucial role in the EMT of CXCR7-overexpressed cells. The above results were also supported by immunofluorescence results (Fig. 3H). Moreover, knockdown of Smad2/3 suppressed invasion and migration in CXCR7-overexpressed cells (Fig. 3I,J). Taken together, these findings indicate that CXCR7 overexpression-induced EMT of HNSCC cells is mediated by TGF-β1-activated Smad2/3 signaling.

### Inhibition of CXCR7 suppresses cell migration and invasion by downregulation of TGF-β1/Smad2/3 signaling

**Figure 4:**
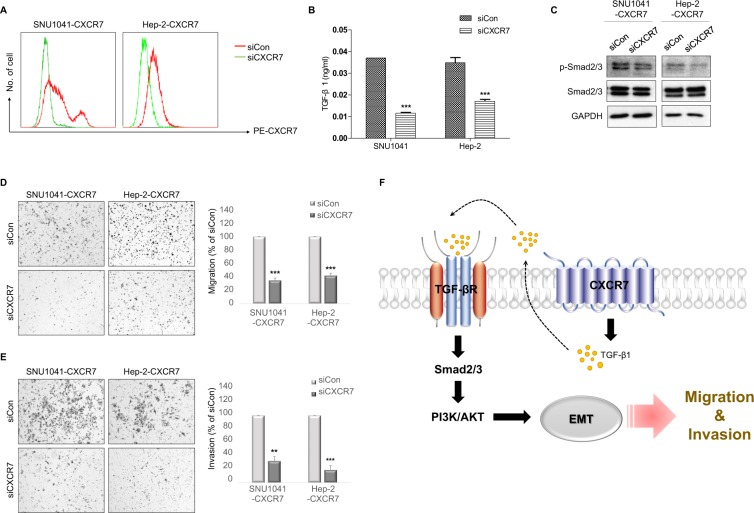
Inhibition of CXCR7 suppressed cell migration and invasion by downregulating TGF-β1/Smad2/3. (**A**) FACS analysis showing that expression of CXCR7 cells was reduced by transfection with CXCR7 siRNA. (**B**) ELISA of TGF-β1 in supernatants of HNSCC cells transfected with CXCR7 siRNA or control siRNA 48 h after culture. (**C**) Western blot analysis showing decreased expression of p-Smad2/3 in CXCR7 knockdown cells compared with control cells. Full-length blots are presented in Supplementary Fig. S3. (**D**,**E**) Transwell migration and invasion assay showing that knockdown of CXCR7 inhibited CXCR7-induced cell migration and invasion. (**F**) Schematic illustration of the CXCR7-mediated regulatory network responsible for the progression of HNSCC. **P < 0.01; ***P < 0.001.

To confirm whether CXCR7 overexpression induces the migration and invasion of HNSCC cells by modulation of TGF-β1/Smad2/3 signaling, we knocked down CXCR7 expression using siRNA in CXCR7-overexpressed cells (Fig. 4A). Knockdown of CXCR7 significantly decreased TGF-β1 levels in conditioned medium (Fig. 4B). Furthermore, knockdown of CXCR7 decreased the phosphorylation of Smad2/3 (Fig. 4C) and significantly inhibited cell migration (Fig. 4D) and invasion (Fig. 4E). These results indicate that CXCR7 overexpression up-regulates cell motility and invasiveness through the canonical TGF-β1/Smad2/3 pathway in autocrine and paracrine manners.

## Discussion

In this study, we demonstrated that CXCR7 is involved in TGF-β1-mediated EMT in HNSCC, resulting in tumor progression (Fig. 4F). Given the demonstrated importance of CXCR7 in tumorigenesis, we examined the expression of CXCR7 in human HNSCC tissues. CXCR7 was differentially expressed in HNSCC cells, and high expression of CXCR7 was associated with aggressive behavior of HNSCC. Overexpression of CXCR7 promoted HNSCC cell metastasis, both *in vitro* and *in vivo*. More importantly, we showed that CXCR7 regulated HNSCC cells by increasing autocrine TGF-β1 signaling, suggesting that CXCR7 could be a potential therapeutic target and predictive indicator in HNSCC.

HNSCC is characterized by high proliferation and regional lymph node metastasis with poor clinical outcome^17,18^. Lymph node metastasis is a well-known prognostic factor in HNSCC^19^. EMT transforms epithelial cells into mesenchymal cells, enabling the migration of cancer cells essential for metastasis^20^. Lymph node metastasis increases the expression of EMT-related proteins, especially in the rapidly growing margin of the lymph node^21^. HNSCC cells that have undergone EMT are more aggressive. Furthermore, loss of E-cadherin associated with EMT is an indicator of poor clinical outcome in HNSCC^22^. In our study, CXCR7 induced an EMT-like phenotype and expression of EMT-related proteins in HNSCC, and it was significantly correlated with lymph node metastasis, large tumor size, and an advanced tumor stage.

The mechanisms involved in the correlation between CXCR7 and poor prognosis have been examined in various cancers. Overexpression of CXCR7 results in increased migration and invasion^23–25^. Stromal cell-derived factor-1α (SDF-1α or CXCL12) is a CXC chemokine that acts as a ligand for two types of receptors, CXCR4 and CXCR7^26^. A recent study showed that SDF-1α binds to CXCR7 10-fold more than it does to CXCR4, and is involved in cell survival, adhesion, and tumor development^27^. Interestingly, CXCR7 overexpression alone increased migration and invasion ability, independently of SDF-1α^28^. In our study, CXCR7 overexpression promoted cell motility, migration, and invasion *in vitro*, and induced lymph node metastasis *in vivo*, independently of SDF-1α. SDF-1α treatment also increased cell migration and invasion (data not shown). Several lines of evidence suggest that PI3K/AKT activity is important for migration and invasion in HNSCC^29,30^. Similarly, we found that CXCR7 overexpression increased PI3K/AKT phosphorylation and promoted the migration and invasion of HNSCC cells. In addition, inhibition of PI3K/AKT suppressed the enhancement of cell migration and the ability to invade, which was induced by CXCR7 overexpression. Moreover, suppression of PI3K/AKT with a PI3K inhibitor resulted in the reversal of EMT marker expression.

TGF-β acts as an early tumor suppressor in tumorigenesis. However, TGF-β becomes a major inducer of EMT during cancer progression in late-stage tumors. It even leads to tumor progression and metastasis by increasing the production of TGF-β in either an autocrine or paracrine manner^31^. In various cancers, TGF-β signaling plays a necessary role in the extracellular microenvironment and cellular mechanisms that promote invasion, migration, proliferation, differentiation, and apoptosis^32^. TGF-β signaling is activated through two main pathways, the canonical or non-canonical. An inactive TGF-β cytokine, which is a latent TGF-β, is located in the extracellular matrix (ECM)^33,34^. In the canonical manner, TGF-β signals via TGF-βRI phosphorylate the cytoplasmic Smad2/3 proteins, which then form a complex with Smad4^35^. This complex regulates transcription of EMT genes^36^. Moreover, TGF-β1 upregulates CXCR7 expression in a Smad2/3-dependent manner in cancer^5,37^. Although some studies have examined the upregulation of CXCR7 by TGF-β1, there has been no research showing that the expression of CXCR7 increases the expression and secretion of TGF-β1. Our study showed that TGF-β1 expression and secretion were induced by CXCR7 overexpression. Notably, CXCR7 overexpression was significantly associated with induction of Smad2/3, PI3K/AKT, MMP-2, and MMP-9 through TGF-β1 activation.

Malignant epithelial cells and stromal cells in the tumor microenvironment produce MMPs and zinc-dependent proteases. Degradation of the ECM is essential for migration and invasion of cancers. Thus, expression of proteases such as MMPs is important for HNSCC invasion^38^. In addition to the pro-tumorigenic function, MMPs have been proposed to be crucial components in the stromal activation of latent TGF-β^39,40^. We wanted to understand how CXCR7 induces TGF-β1 production, leading to EMT. Our data show that CXCR7 overexpression induced activation of MMP2 and MMP9. This finding suggests that CXCR7-mediated activation of MMP2 and MMP9 induces the activation of latent TGF-β1 and ECM degradation.

TGF-β-induced EMT has been known to transmit through both Smad- and non-Smad-dependent signals, involving crosstalk between PI3K/AKT and Smad proteins^41,42^. According to our findings, CXCR7 overexpression enhanced TGF-β1-induced cell migration, invasion, and EMT. CXCR7 induced the activation of PI3K/AKT but not of MAPKs including ERK, JNK, and p38 MAPK. Furthermore, inhibition of TGF-β signaling with siRNA for Smad2/3 suppressed activation of PI3K/AKT and restored EMT marker expression. However, suppression of PI3K/AKT with a PI3K inhibitor did not influence Smad2/3 phosphorylation (data not shown). These results imply that CXCR7 partially activates PI3K/AKT through TGF-β mediated Smad2/3 activation. Thus, we demonstrated that CXCR7 is a master regulator of oncogenic TGF-β1/Smad2/3 signaling in HNSCC.

In conclusion, we demonstrated that CXCR7 plays a key role in the progression of HNSCC. Overexpression of CXCR7 in HNSCC was significantly associated with tumor metastasis. Furthermore, functional and mechanistic studies revealed that CXCR7 regulated EMT and ECM remodeling to achieve higher motility and invasiveness through the activation of TGF-β1/Smad2/3 signaling. Therefore, our study suggests that CXCR7 may act as an effective prognostic marker and a promising treatment target in HNSCC.

## Methods

### Patients and tumor samples

A total of 103 HNSCC tissues were recruited from the Chungnam National University Hospital. All samples used in the study were approved by the ethics committee of Chungnam National University Hospital. Written informed consent was obtained from all the patients. Clinicopathologic characteristics were estimated, including gender, age, stage, lymph node metastasis, and tumor invasion. Patients were staged according to the tumor node metastasis (TNM) staging system, and all samples were confirmed by pathological analysis. This study was conducted in accordance with the Helsinki Declaration and Good Clinical Practice.

### Tissue microarray construction

Tissue microarray (TMAs) construction was performed using our previously reported protocols^43^. Tissue microarrays were constructed from 103 cases of archived formalin-fixed and paraffin-embedded tissue blocks from HNSCC patients. For each tumor, a representative tumor area was carefully selected from a hematoxylin and eosin (H&E)-stained section of a donor block. Each case was represented by two cylindrical cores (2-mm diameter) from a tumor, which was punched using an automated tissue arrayer (UNITMA, Seoul, South Korea). Tissue microarray blocks containing 206 cylinders were constructed.

### Specimen preparation and immunohistochemistry

Specimen preparation and immunohistochemistry was conducted as previously described^43^. For immunohistochemistry, 3-μm-thick sections were cut from the recipient blocks. All procedures were performed at room temperature, as recommended by the manufacturer. In brief, sections were dewaxed in xylene and then rehydrated in graded alcohols. Sections were washed in water before antigen retrieval using a Dako PTLink machine (Dako, Glostrup, Denmark) with 10 mM sodium citrate buffer (pH 6.0) at 97 °C for 20 min. Sections were treated with 3% hydrogen peroxide for 10 min to block endogenous peroxidase and preincubated with a serum-free protein block solution (Dako) for 20 min to eliminate background staining. Prepared polyclonal mouse antibodies raised against human CXCR7 (Abcam, Cambridge, UK; ab38089) and TGF-β1 (ABcam; ab169771) were diluted at 1:1000 with background-reducing diluents (Dako). Samples were incubated overnight at 4 °C in a humidified chamber and washed with TBS-T. Slides were then incubated for 30 min with an EnVision anti-mouse (Dako) polymer. Reaction products were visualized with diaminobenzidine (DAB) plus substrate–chromogen solution for 5 min. Slides were counterstained with Meyer’s hematoxylin and mounted. Careful rinses with several changes of phosphate buffered saline (PBS) were performed between stages of the procedure. Negative controls consisted of excluding the primary antibody or using preimmune IgG1 to evaluate nonspecific staining.

### Evaluation of immunohistochemical staining

Evaluation of immunohistochemical staining was conducted as previously described^43^. Immunohistochemical staining results were evaluated by two independent pathologists who were blinded to the patients’ clinicopathological details. The immunohistochemical staining was categorized according to the following scoring method, which included four grades based on staining intensity: 0, no staining; 1, weak staining; 2, moderate staining; and 3, strong staining. In the case of heterogeneous staining within samples, the higher score was selected if more than 50% of cells showed higher staining intensity. For all patients, scores from two tumor cores in the same patient were averaged to obtain a mean score. Cases with staining intensity scores of 0–1 were placed in the CXCR7 low-expression group, and those with staining intensity scores of 2–3 were placed in the CXCR7 high-expression group.

### Cell culture

The human HNSCC cell lines SNU1041 (KCBL No.01041) and Hep-2 (KCBL No.10023) were purchased from the Korean Cell Line Bank (Seoul, South Korea). SNU1041 and Hep-2 were maintained in RPMI1640 and DMEM (Welgene, Daegu, South Korea) supplemented with 10% fetal bovine serum (FBS) and 1X penicillin/streptomycin (Welgene), respectively. Cells were cultured at 37 °C under 5% CO_2_ in a humidified incubator.

### Reagents and antibodies

Antibodies against CXCR7 (ABcam; ab38089), GAPDH (Santa Cruz Biotechnology, Dallas, TX, USA; sc-25778), E-cadherin (Cell Signaling Technology, Danvers, MA, USA; 3195), Ep-CAM (Santa Cruz Biotechnology; sc-25308), N-cadherin (Cell Signaling Technology; 13116), α-smooth muscle actin (Sigma Aldrich, St. Louis, MO, USA; A5228), Slug (Cell Signaling Technology; 9585), Twist (Santa Cruz Biotechnology; sc-81417), Vimentin (Cell Signaling Technology; 3932), phosphorylated-AKT at Ser^473^ (Cell Signaling Technology; 9271), AKT (Cell Signaling Technology; 9272), phosphorylated-ERK1/2 (Cell Signaling Technology; 9101), ERK1/2 (Cell Signaling Technology; 9102), phosphorylated-JNK (Cell Signaling Technology; 4668), JNK (Cell Signaling Technology; 9251), phosphorylated-p38 (Cell Signaling Technology; 9211), p38 (Cell Signaling Technology; 9212), TGF-β1 (Cell Signaling Technology; 3711), phosphorylated-Smad2/3 (Cell Signaling Technology; 8828), Smad2/3 (Cell Signaling Technology; 8685), MMP2 (ABcam; ab37150), and MMP9 (ABcam; ab38898) were used in Western blotting and immunofluorescence. Small interfering (si) RNAs for controls, CXCR7, and Smad2/3 were purchased from Santa Cruz Biotechnology and Thermo Fisher Scientific (St. Louis, MO, USA). For inhibition of protein kinases, LY294002 and wortmannin were purchased from Sigma Aldrich.

### CXCR7 overexpression in HNSCC cell lines

CXCR7 overexpression in HNSCC cell lines was conducted as previously described^44^. Overexpression of CXCR7 in HNSCC cells was achieved through the use of lentivirus-mediated transduction of full-length human CXCR7 subcloned into a pLVX-EF1 α-IRES-Puro lentiviral vector (Clontech, Mountain View, CA, USA). To generate a stable transfectant, the acquired lentiviral vector was co-transfected into 293 T cells with virus packaging mix (Sigma Aldrich) using a Lipofectamine 3000 (Invitrogen) according to the manufacturer’s protocol. The virus was harvested from the supernatant and concentrated with lenti-X-concentrator (Clontech), then added to SNU1041 and Hep-2 cells along with 5-µg/mL polybrene (Santa Cruz). Puromycin-resistant cells were selected by culture for 2 weeks in the presence of puromycin. CXCR7 expression levels were analyzed by flow cytometry (Beckman Coulter, Brea, CA, USA) and Western blot analysis.

### siRNA transfection

We obtained the CXCR7 siRNAs (CGC UCU CCU UCA UUU ACA, Bioneer, Daejeon, Korea), pre-made Smad2/3 siRNA (Santa Cruz) and negative control siRNA (Santa Cruz). Cells were transfected using Lipofectamine RNAiMax (Invitrogen, Carlsbad, CA, USA).

### Wound healing assay

The wound healing assay was performed as previously described^44^. Briefly, 5 × 10^4^ cells were seeded on each side of the chamber (Ibidi, Munich, Germany) with culture inserts for live-cell analysis. After suitable cell growth for 24 h, the culture-inserts were detached, and cells were incubated with fresh culture medium. Cells were monitored over a 24 h period.

### Migration and invasion assays

Migration and invasion of HNSCC cells were implemented using a 8-µm pore size transwell chamber (Corning Costar, Cambridge, MA). Migration and invasion assays were performed using previously reported protocols^44^. Briefly, the lower surface of the transwell was coated with 0.1% gelatin (Sigma Aldrich) for the migration assay, and the upper side was coated with 25 µg/ml of matrigel for the invasion assay (Matrigel; BD Biosciences, Franklin Lakes, NJ, USA). Fresh culture medium containing 10% FBS was placed in the lower chamber as a chemo-attractant. HNSCC cells were suspended at a final concentration of 1 × 10^5^ cells/ml in medium containing 0% FBS. Cell suspensions were loaded into each of the upper wells, and the chamber was incubated at 37 °C for 24 h (migration) or 48 h (invasion). Cells were fixed and stained with 0.1% crystal violet staining. Chemotaxis activity was quantified by counting the cells that migrated to the lower side of the filter with a microscope. Five randomly chosen fields were counted for each assay.

### Western blot analysis

Western blotting was performed using our previously reported protocols^44^. Briefly, cells were lysed in ProEX^TM^ CETi Lysis buffer (TransLab, South Korea) with protease inhibitor cocktail (Sigma Aldrich) and phosphatase inhibitor cocktail (Roche). Cell lysates were separated using SDS-PAGE and then transferred to polyvinylidene difluoride (PVDF) membranes (PALL). The membranes were incubated with the indicated primary antibodies, followed by incubation with horseradish peroxidase-conjugated secondary antibodies (Cell Signaling Technology). The immunoreactive polypeptides were detected using an ECL substrate (Bio-rad and Thermo Fisher Scientific).

### Immunofluorescence

Immunofluorescence was performed using our previously reported protocols^44^. Briefly, cells were attached to a chamber slide^TM^ (Lab-TekII). After starvation for 6 h in serum-free medium, cells were fixed in 10% formalin for 10 min at 37 °C, permeabilized with 0.5% Triton X-100 (Sigma Aldrich) for 20 min at room temperature, washed in PBS, and then blocked in 3% chicken serum albumin (CSA) in PBS for 30 min at room temperature. Cells were incubated with anti-E-cadherin, anti-Vimentin and anti-CXCR7, respectively, overnight at 4 °C. The primary antibody was removed and washed and then treated with a fluorescence-conjugated secondary antibody for 2 h at 37 °C. Cells were stained with DAPI (Vector Laboratories, Burlingame, CA, USA) for nuclei staining, and coverslips were mounted on the slides.

### Orthotopic model of head and neck cancer

All animal experiments were approved by the Animal Experimental Ethics Committee of Chungnam National University and animal care was performed in accordance with the guideline. Head and neck tumors in mice were generated by modifying orthotopic xenograft models as previously described by Kawashiri *et al*. and Sano *et al*.^45,46^. Inoculation with SNU1041-mock and SNU1041-CXCR7 cells was performed in five mice. In preparation for inoculation, cells were resuspended in RPMI1640 at a concentration of 1 × 10^7^ cells/100 μl. Inoculation was performed by injecting 20 µl of cell suspension to deliver 2,000,000 cells into the submucosa of the dorsal tongue at the circumvallate line. The animals were euthanized 8 weeks after inoculation and the cervical lymph nodes were removed by microsurgical dissection. Specimens were formalin-fixed, paraffin-embedded, serially sectioned on 200 µm sections, and stained with H&E.

### Enzyme-linked immunosorbent assay analysis

Conditioned medium from each group was collected and stored at −80 °C before enzyme-linked immunosorbent assay (ELISA) analysis. The active form TGF-β1 was measured using a human TGF-β1 ELISA kit (B&D Systems) according to the manufacturer’s instructions.

### Gelatin zymography assay

The gelatin zymography assay was previously described by HW Yeh *et al*.^47^. Conditioned medium from each group was collected and concentrated using a centricon (Pall Corporation, 30 K). Samples were mixed with 2X sample buffer (TransLab, South Korea) and separated by 8% SDS-PAGE containing 0.1% gelatin (Sigma Aldrich). After electrophoresis, gels were washed two times in washing buffer (2.5% Triton-X 100 in TBS) and then incubated overnight at 37 °C in 1X zymogram development buffer (Bio-Rad; 161-0766). After incubation, the gels were washed and stained with EZBlue^TM^ Gel Staining Reagent (Sigma Aldrich; G1041) and then washed until white bands appeared on the blue background.

### Statistical analysis

The expression analysis was performed using Gene Expression Profiling Interactive Analysis (GEPIA). Based on TCGA Datasets, the GEPIA website (http://gepia.cancer-pku.cn/) was used for correlation between CXCR7 and TGF-β1. The χ^2^ test and linear-by-linear association were used to assess correlations between CXCR7 expression and clinicopathological features. All analyses were conducted using the SPSS version 17.0 software program (SPSS, Chicago, IL). Data from *in vitro* and *in vivo* experiments were expressed as the mean ± standard error of the mean (SEM). Differences between groups were analyzed using the Student’s t-test. A *P*-value < 0.05 was considered to indicate statistical significance. Data are representative of at least three independent experiments.

## Supplementary information

Supplementary Information

## Data Availability

The datasets generated and/or analyzed during the current study are available from the corresponding author on reasonable request.
