# Supplementary material for: CXCR7 promotes migration and invasion in head and neck squamous cell carcinoma by upregulating TGF-β1/Smad2/3 signaling
Source: Sci Rep. 2019 Dec 2;9:18100. doi: 10.1038/s41598-019-54705-x (PMC6889124; doi:10.1038/s41598-019-54705-x)
Supplement: Supplementary file 1 — Supplementary Information [file 41598_2019_54705_MOESM1_ESM.docx]

**Supplementary Information**

**CXCR7 promotes migration and invasion in head and neck squamous cell carcinoma by upregulating TGF-β1/Smad2/3 signaling**

Nayoung Kim, Hyewon Ryu, Solbi Kim, Mina Joo, Heung Jin Jeon, Myung-Won Lee, Ik-Chan Song, Mi-Na Kim, Jin-Man Kim, Hyo Jin Lee


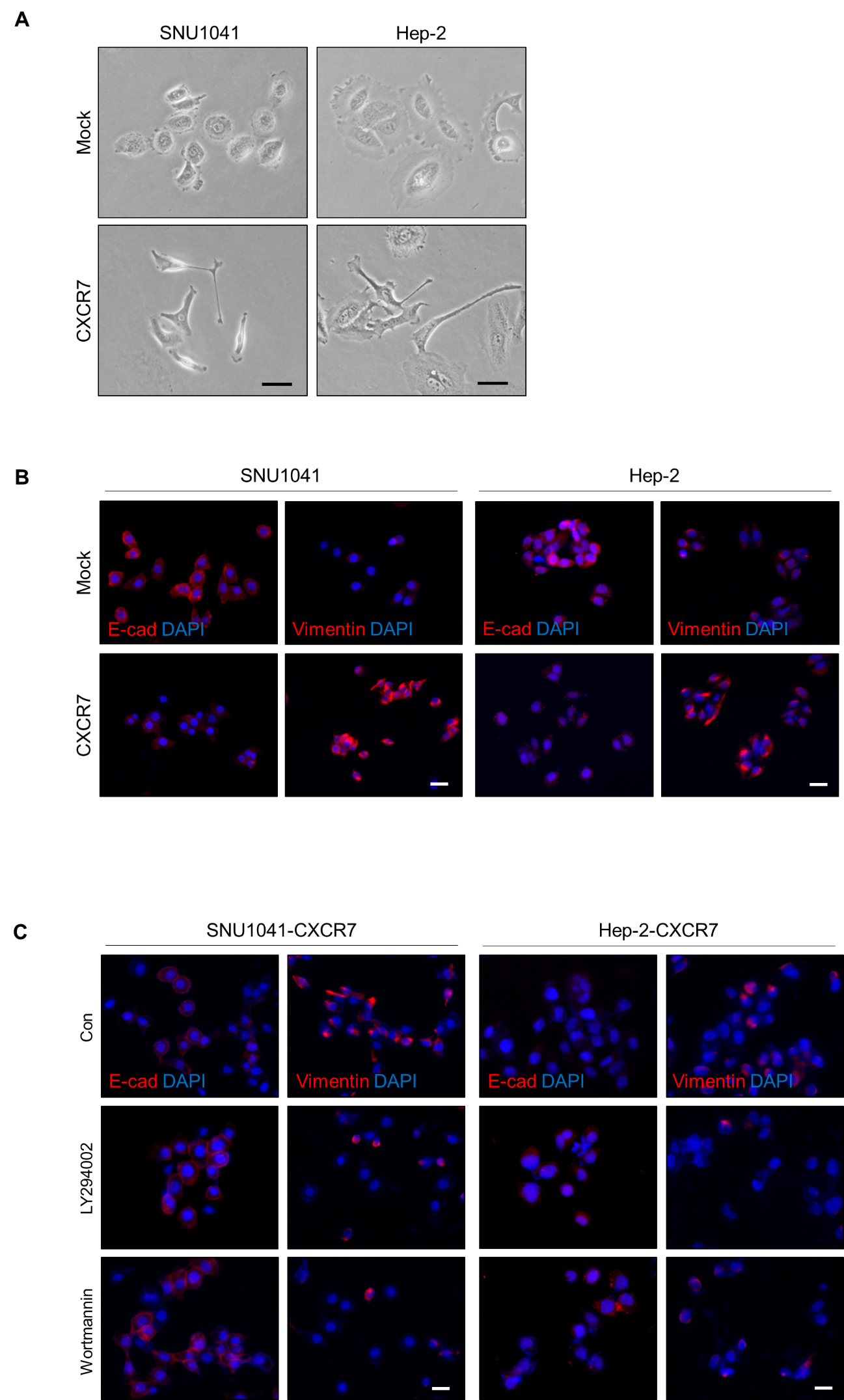


**Supplementary Figure S1**. CXCR7 overexpression enhanced EMT through the AKT signaling pathway. A. Morphological changes after overexpression of CXCR7 in HNSCC cells. Scale bars, 50 μm. B. Immunoﬂuorescence staining showing reduced expression of E-cadherin and upregulated expression of vimentin after CXCR7 overexpression. Scale bars, 20 μm. C. Immunoﬂuorescence staining showing increased expression of E-cadherin and downregulated expression of vimentin induced by PI3K/AKT inhibition. Scale bars, 20 μm. Refer to Figure 2.


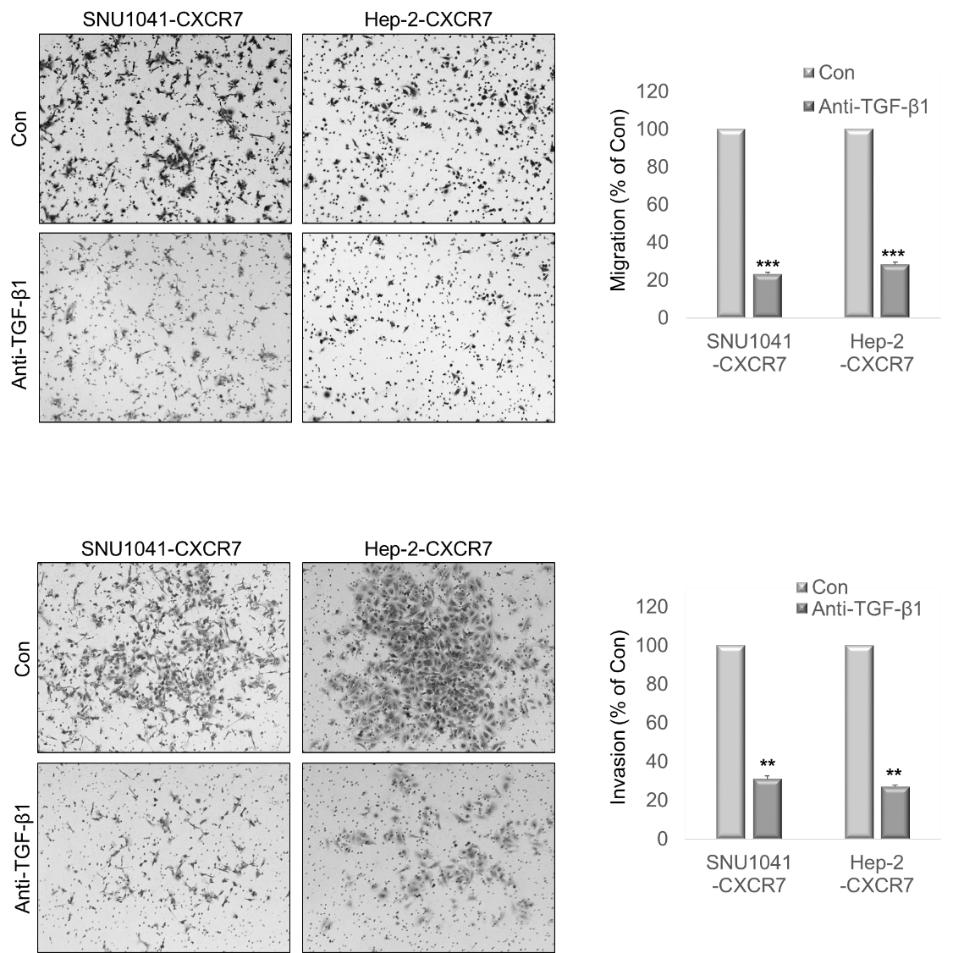


**Supplementary Figure S2.** Neutralization of secreted TGF- β1 suppressed cell migration and invasion in CXCR7-overexpressed cells. Migration and invasion assay were performed with Transwell chamber. Con, Control; Anti-TGF-β1, Anti-TGF-β1 monoclonal antibody (0.5 μg/ml). **, *P* < 0.01; ***, *P* < 0.001. Refer to Figure 3.


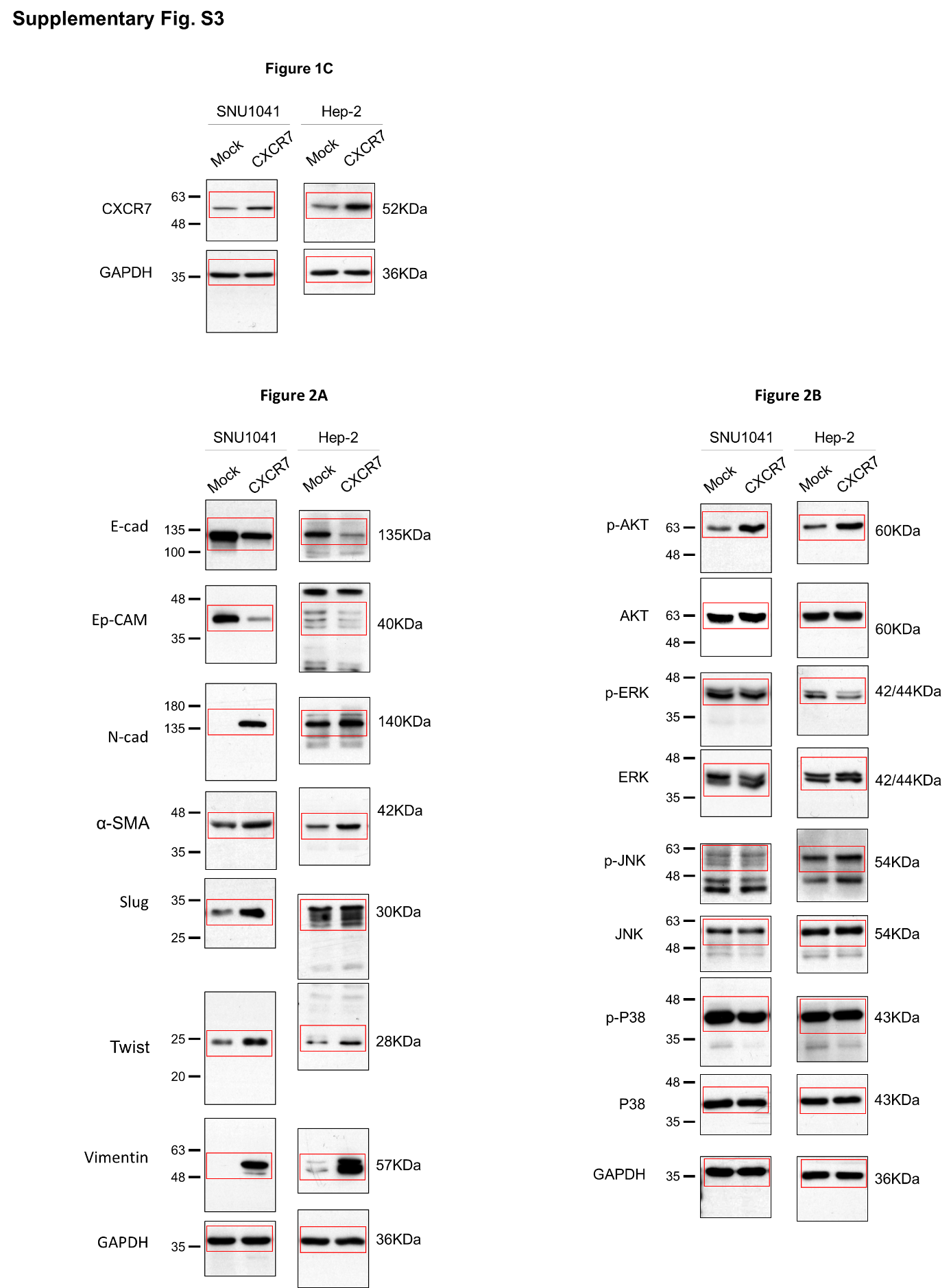


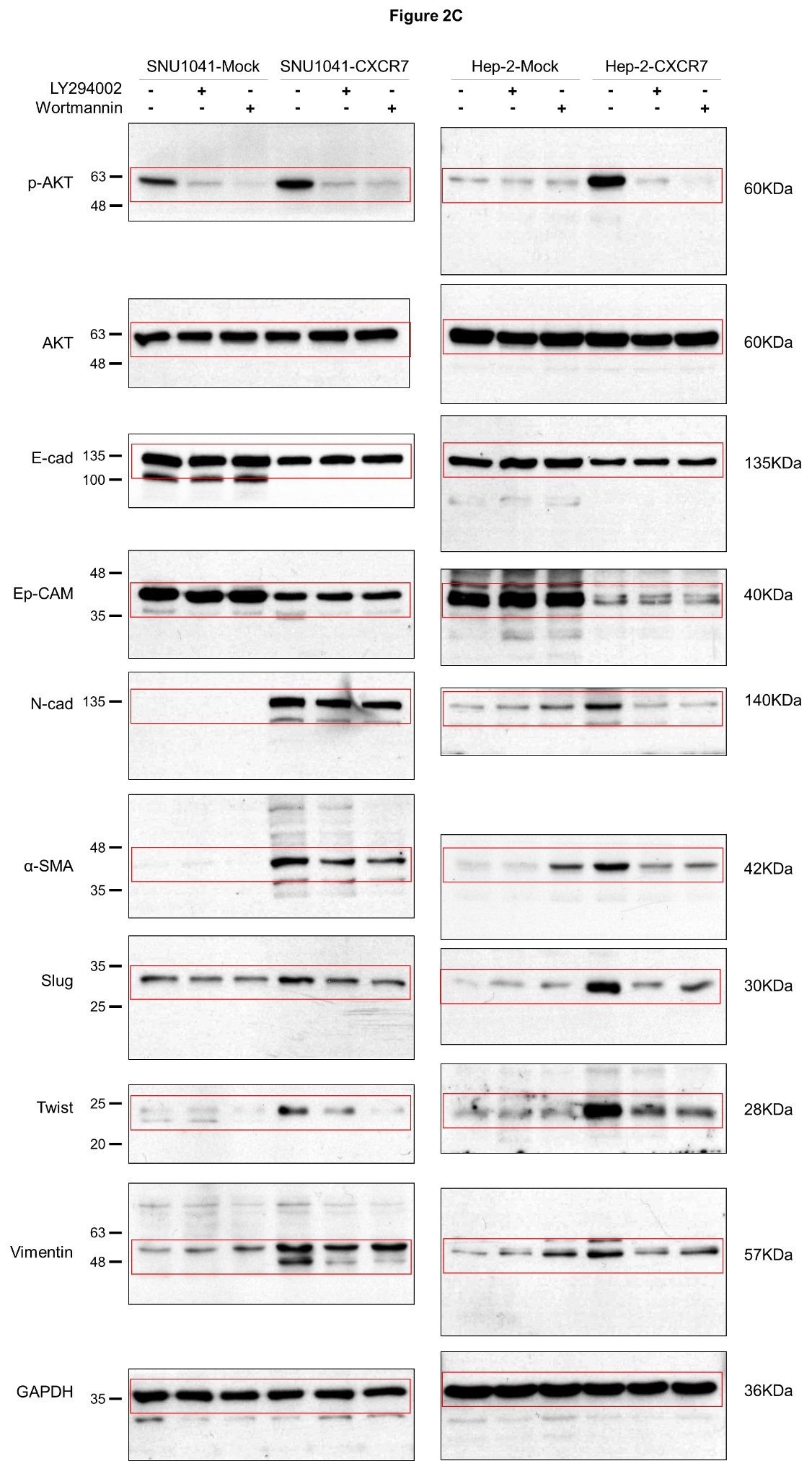


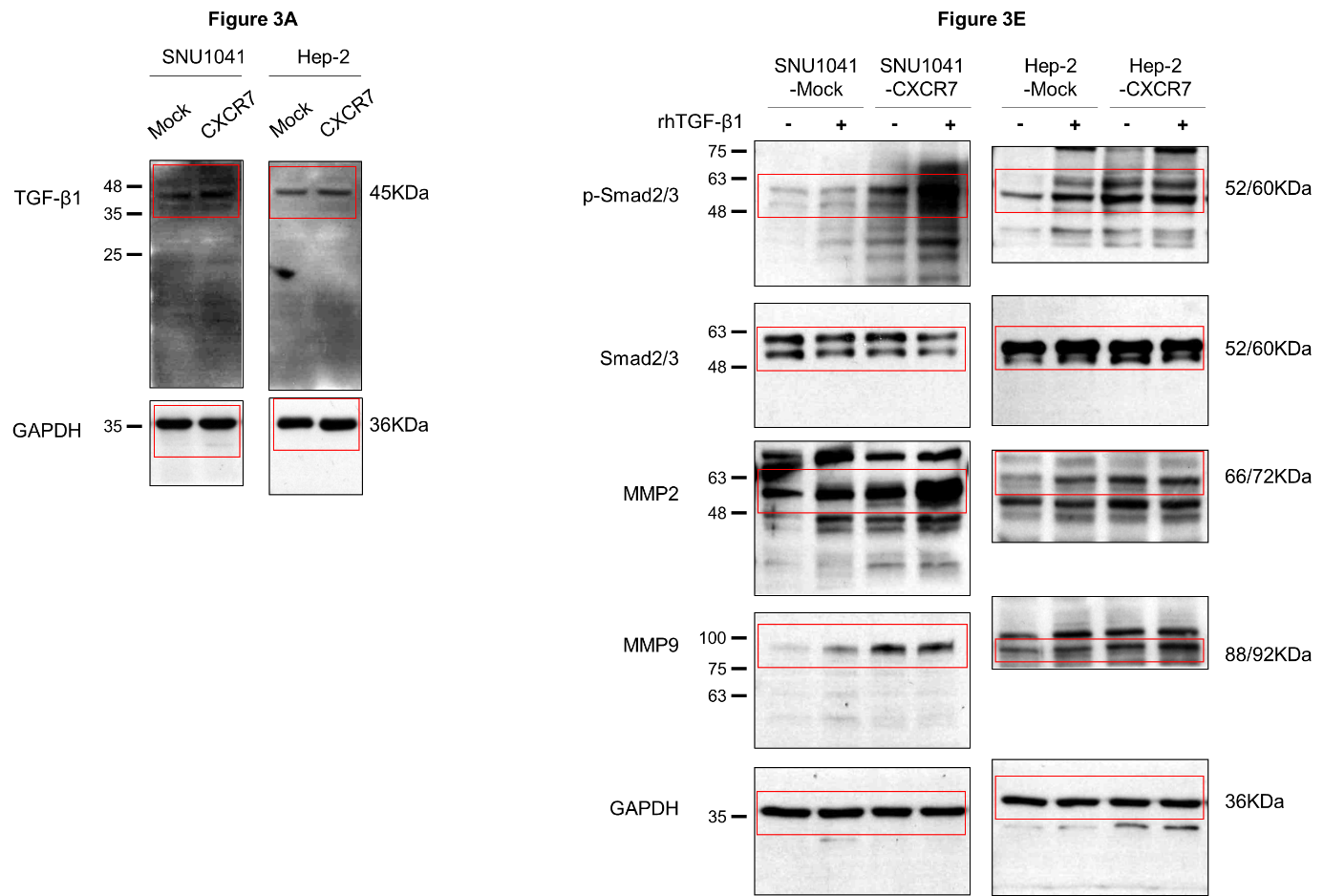


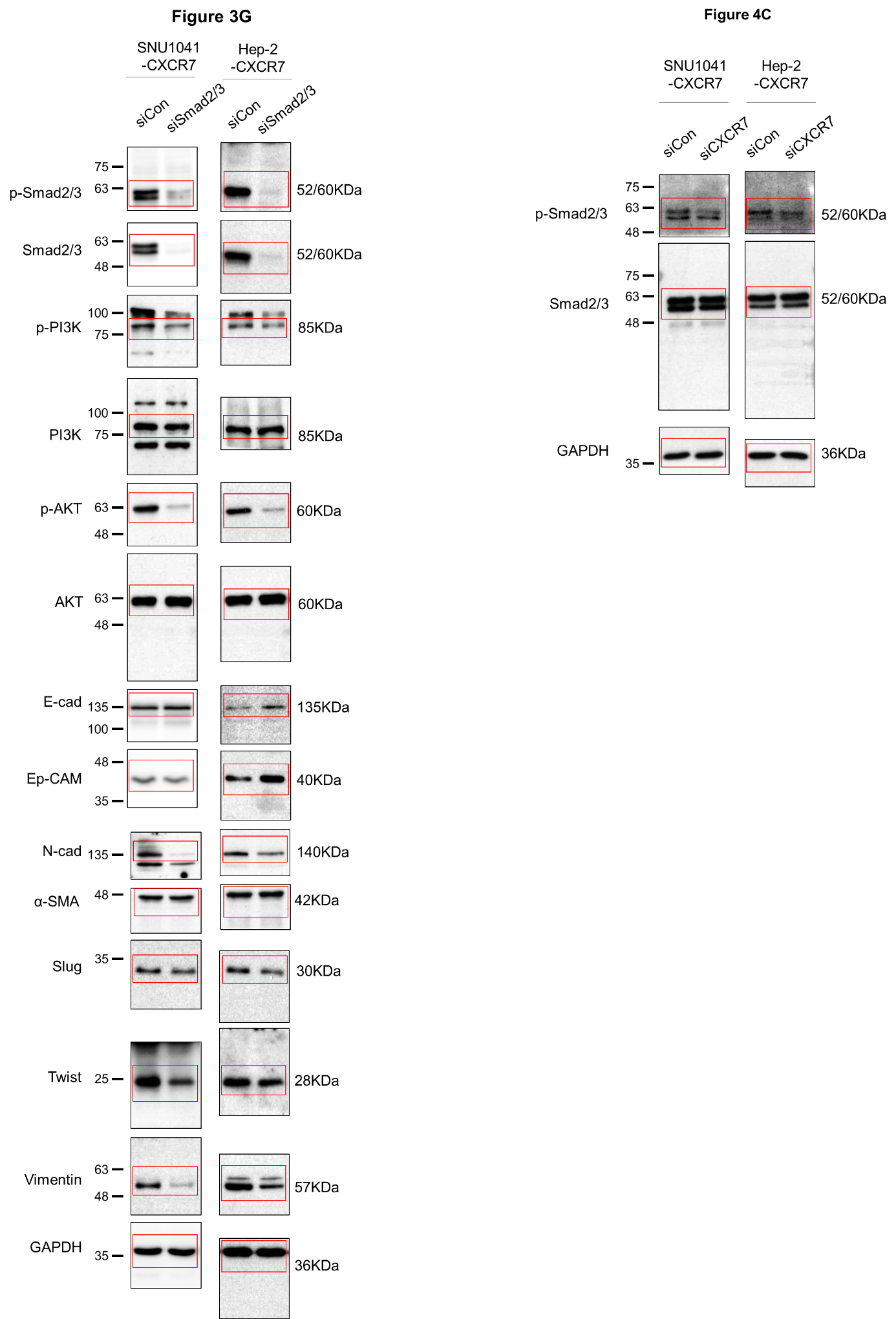


**Supplementary Figure S3**. Uncropped scans of western blots displayed in Fig. 1C, 2A, 2B, 2C, 3A, 3E, 3G and 4C.
